# A novel intradermal tattoo-based injection device enhances the immunogenicity of plasmid DNA vaccines

**DOI:** 10.1038/s41541-022-00581-y

**Published:** 2022-12-22

**Authors:** Alejandro M. Gomez, George (Giorgi) Babuadze, Marc-André Plourde-Campagna, Hiva Azizi, Alice Berger, Robert Kozak, Marc-Antoine de La Vega, Ara XIII, Maedeh Naghibosadat, Marie-Edith Nepveu-Traversy, Jean Ruel, Gary P. Kobinger

**Affiliations:** 1 Département de Microbiologie-Infectiologie et Immunologie, Faculté de Médecine, Université Laval, Québec, QC G1V 0A6 Canada; 2 Biological Sciences Platform, University Toronto, Sunnybrook Research Institute at Sunnybrook Health Sciences Centre, Toronto, ON Canada; 3 Département de Génie Mécanique, Université Laval, Québec, QC G1V 0A6 Canada; 4 Department of Microbiology and Immunology, University of Texas Medical Branch, 301 University Blvd, Galveston, TX 77555 USA; 5 Global Urgent and Advanced Research and Development (GuardRX), Batiscan, QC G0X 1A0 Canada

**Keywords:** DNA vaccines, DNA vaccines

## Abstract

In recent years, tattooing technology has shown promising results toward evaluating vaccines in both animal models and humans. However, this technology has some limitations due to variability of experimental evaluations or operator procedures. The current study evaluated a device (intradermal oscillating needle array injection device: IONAID) capable of microinjecting a controlled dose of any aqueous vaccine into the intradermal space. IONAID-mediated administration of a DNA-based vaccine encoding the glycoprotein (GP) from the Ebola virus resulted in superior T- and B-cell responses with IONAID when compared to single intramuscular (IM) or intradermal (ID) injection in mice. Moreover, humoral immune responses, induced after IONAID vaccination, were significantly higher to those obtained with traditional passive DNA tattooing in guinea pigs and rabbits. This device was well tolerated and safe during HIV vaccine delivery in non-human primates (NHPs), while inducing robust immune responses. In summary, this study shows that the IONAID device improves vaccine performance, which could be beneficial to the animal and human health, and importantly, provide a dose-sparing approach (e.g., monkeypox vaccine).

## Introduction

Intradermal (ID) delivery of vaccines is one of the first vaccination routes known to confer protection to recipients (e.g., variola vaccine^1^). Since then, this delivery method has been extensively evaluated within over 200 clinical trial studies^2,3^. Analyses of these studies have concluded that ID delivery can generate comparable or superior immune responses to other vaccination sites (e.g., intramuscular), leading to dose-sparing and improved immune stimulation^2,4,5^. Major drawbacks associated with ID immunization include the technical difficulty to inject within the intradermal space (and not subcutaneous) and the limited volume of vaccine that can be delivered in the ID space (0.1 ml in humans per injection). Many of the currently licensed vaccines are delivered with a unique syringe and needle through traditional routes of immunization, such as intramuscular (IM), oral, or subcutaneous injections; and for many vaccines, multiple injections over time, or boosts, are required to generate durable and protective immune responses^6^.

In recent years, new routes of administration for vaccines have been studied with the objective of increasing their immunogenicity, thereby reducing the number of injections needed to generate a protective immune response against a specific pathogen. In particular, intradermal (ID) delivery of DNA vaccines generates broad immune responses and protects against pathogens with a reduced number of vaccination doses^2^. The skin is a promising route for the administration of vaccines given that the dermis and epidermis are abundant in immune cells, specifically antigen-presenting cells (APCs). Consequently, data in animal and human trials show that ID administration is superior to traditional administration of vaccines into the muscle or subcutaneous tissue^4^. Currently, the existing devices used for ID vaccination (e.g., ID needle/syringes, gene gun, jet injectors, electroporation (EP), or microneedle patches) can deliver only a small amount of vaccine preparation due to the limited skin area accessible with a needle, small patch, or highly pressurized injection. The maximum volume that can be administrated to humans and most large animal species with a needle (Mantoux protocol) is 0.1 ml per injection. While theoretically, the concentration of vaccine could be increased in this small volume, there are numerous challenges, including the requirement for accommodating the maximum viscosity and self-precipitation of proteins or nucleic acids^7^.

Recently, a new strategy for ID DNA vaccine delivery, named DNA tattooing, has been evaluated in animal models and humans. Data from different studies show that DNA tattooing can elicit vigorous cellular and humoral immune responses against vaccine antigens^8–14^. The benefits of DNA tattooing include the ability to increase the vaccine administration area within the epidermal skin layer compared to traditional intradermal injection, potentially resulting in increased contact between a larger quantity of immune cells and the vaccine. In addition, DNA tattooing involves the administration of vaccine preparations through thousands of skin perforations, causing controlled skin damage, sub-epidermis inflammation and attractant signals^15^, which could act as an adjuvant and increase vaccine immunogenicity. Prior to the current study, investigations concerning DNA tattooing have been performed using traditional tattoo machines, which are not designed to control the volume of vaccine inoculated; the vaccine solution is deposited on the skin surface and then an oscillating tattoo needle is applied at the tattooing site to penetrate the skin (passive migration of the vaccine into the skin). This technique does not allow for complete administration of the vaccine preparation into the skin, and a considerable amount of solution does not penetrate the dermis where APCs are located^16–18^, resulting in the inefficiency of dosage.

To address these limitations, we designed and developed a new vaccine-delivery device capable of safely administering intradermally 1 ml of vaccine in a few seconds. This technology was inspired by traditional tattoo machines and modified to actively inject pressurized vaccine under the dermis at a rate of 100 microinjections per second. We demonstrate here that this new technology improves vaccine delivery, and thus vaccine immunogenicity, when compared to IM and traditional tattooing-delivery methods in mouse, guinea pig, hamster, rabbit, and non-human primate (NHP) models. DNA-based vaccines were selected over other vaccines for their relative low potency and ease of production, thus making the recording of enhanced vaccine potency against several antigens more experimentally practical.

## Results

### DNA tattooing increases immunogenicity when compared to traditional IM and ID injection

Based on previous reports showing that tattoo immunization improves antigen-specific immune responses of several DNA vaccines^8–14^, we tested whether this methodology, using a traditional tattoo machine, could improve the immune responses directed to ZGP in vaccinated mice when compared to traditional IM and ID (Mantoux technique) injections. Cellular immune responses were analyzed 10 days after the last boost. ELISpot analysis showed that passive tattoo immunization induces higher responses of IFN-γ (1030 ± 58 SFU/10^6^ cells) than traditional IM (773 ± 23 SFU/10^6^ cells) and ID (42 ± 14 SFU/10^6^ cells) injections and control group (30 ± 2 SFU/10^6^ cells) on average (Supplementary Fig. S1A). Interestingly, ID immunization induced low ZGP-specific IFN-γ responses that barely increased after the last immunization when compared to the control group. In addition to the cellular immune response, the serum IgG responses to ZGP protein were analyzed by enzyme-linked immunosorbent assay (ELISA). Passive tattoo immunization dramatically increased ZGP-specific IgG levels when compared to traditional routes of immunization (IM and ID) and the control group. On day 84, following boost, vaccinated animals showed an increase in optical density (OD) of 30-fold in the passive tattoo group, 5.3-fold in IM group, and 2.69-fold in ID group, respectively, when compared to control group. Moreover, humoral immune responses continued to increase two weeks after the last immunization with DNA tattooing, while they declined after the last immunization in the IM group. Of note, while IM immunization induced significant cellular immune responses, this increase did not lead to the development of a strong humoral response, as observed with DNA tattooing (Supplementary Fig. S1B).

### Analysis of antigen-encoding RNA expression in skin after plasmid delivery with IONAID

**Fig. 1:**
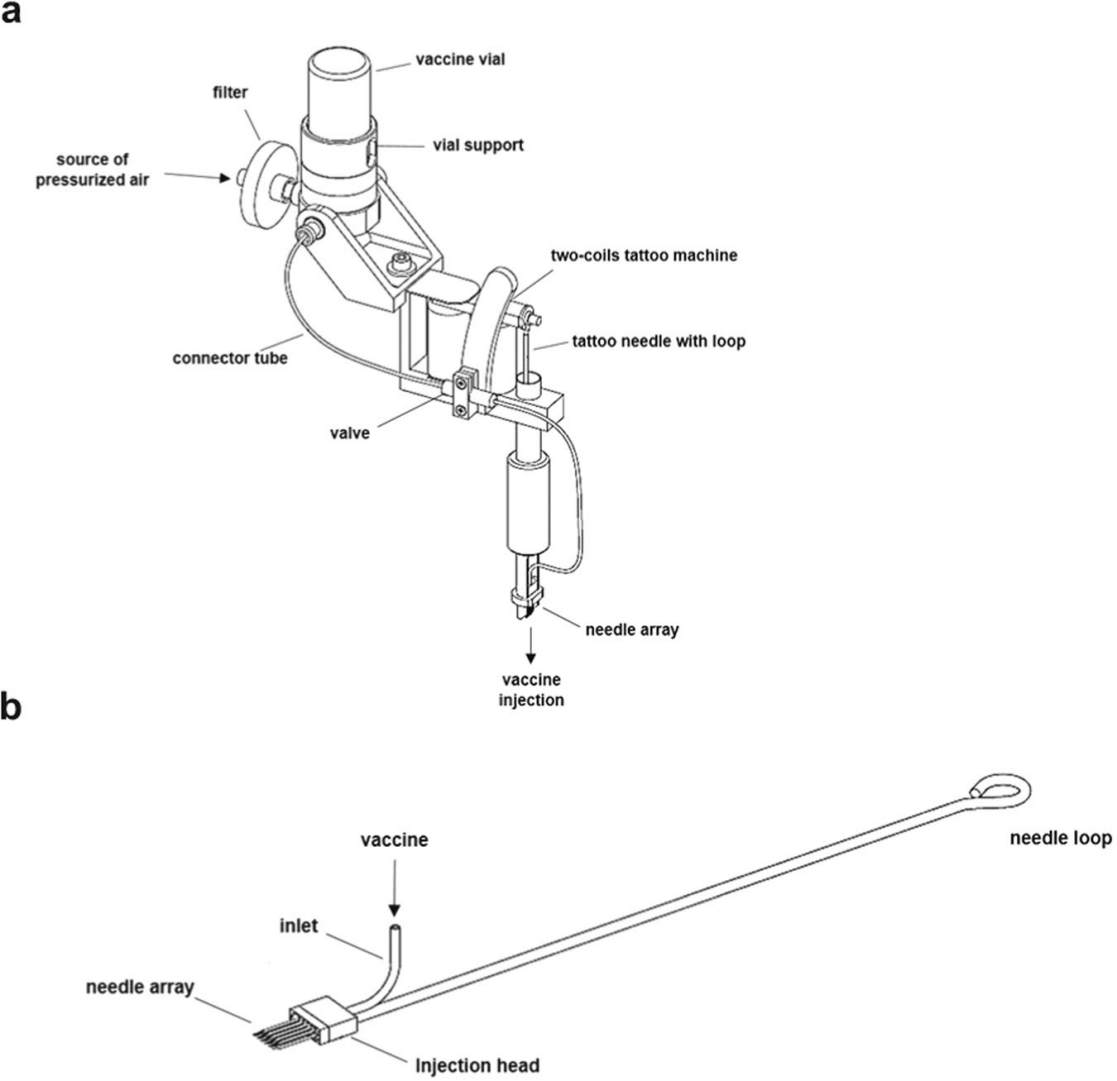
The prototype of the intradermal oscillating needle array injection device (IONAID). **a** Concept drawing showing the main components of IONAID. **b** Close up of a needle array composed of 11 hypodermic needles.

**Fig. 2:**
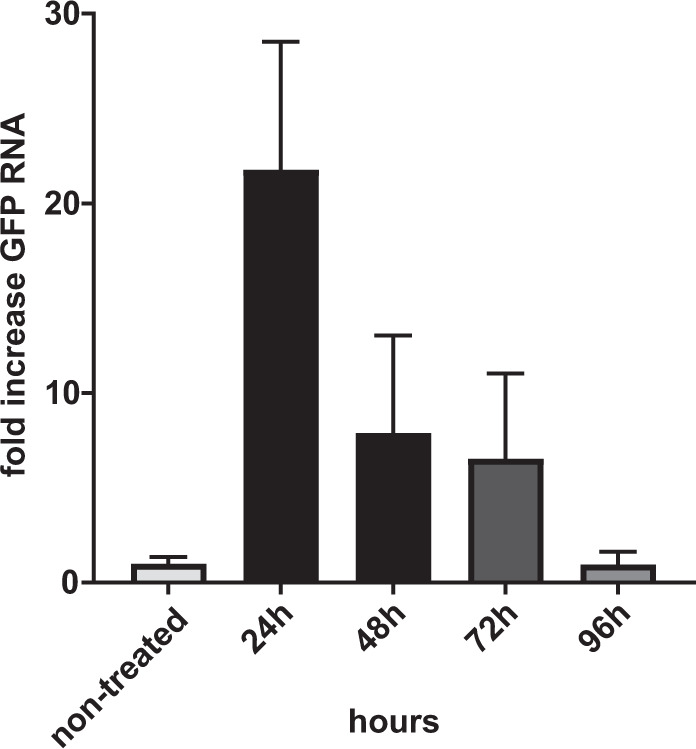
Time course of GFP RNA expression in mouse skin after vaccination using IONAID. In all, 100 µg of reporter gene plasmid coding for eGFP was delivered in the back skin of C57BL/6 mice. Total RNA was extracted from skin specimens and eGFP mRNA was quantified by qPCR at different times post vaccination. Results are expressed as fold increase relative to non-treated skin specimens. Data shown represent the means ± SEM of three animals per group.

DNA tattooing showed promising results in increasing the immunogenicity of pcDNA3.1-EbovZGP plasmid; however, vaccine injection could not be efficiently controlled, and a considerable loss of vaccine solution occurred during the procedure. To improve vaccine delivery into the skin, we developed a new vaccine delivery device, based on the tattoo technology, capable of safely injecting intradermally 1 ml of pressurized vaccine in seconds (Fig. 1a, b). To analyze the performance of this new device to deliver DNA vaccines, a pIDV-II plasmid coding for eGFP was used to monitor the kinetics of antigen expression after DNA tattooing with IONAID. Needle depth was adjusted to 1 mm and the plasmid solution (100 µl at 1 mg/ml) was applied on the back skin of C57BL/6 mice. Skin specimens of the tattoo sites were dissected at 24 h, 48 h, 72 h, and 96 h post vaccination, total RNA was extracted, and GFP RNA levels were analyzed by qPCR. Average levels of GFP RNA increased from the base level by 22-fold at 24 h post vaccination, and then proceeded to decrease to 7.9- and 6.5-fold at 48 h and 72 h post vaccination respectively (Fig. 2). By 96 h post vaccination, levels of GFP expression returned to base values.

### IONAID enhances ZGP-specific immune responses in mice

**Fig. 3:**
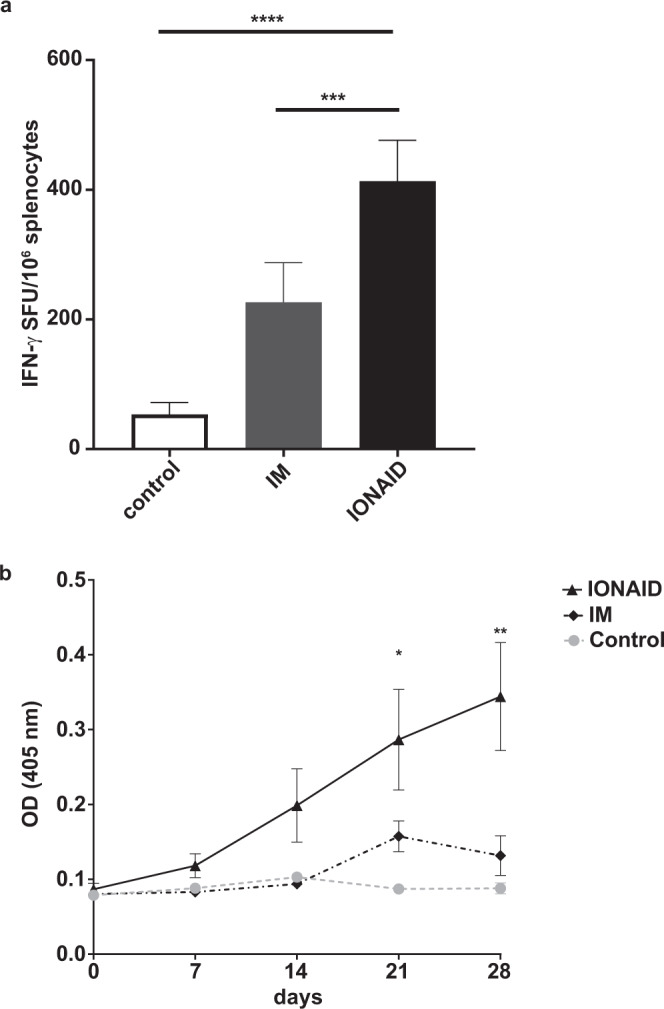
ZGP-enhanced immune responses by IONAID in mice. **a** ZGP-specific IFN-γ responses in groups of C57BL/6 mice (*n* = 3/group) after vaccination with 100 µg of pIDV-II-EboV-GP-M06 as measured by ELISpot assay. Splenocytes were purified at day 10 after immunization and stimulated with a pool of peptides from GP/Mayinga-76 of Zaire Ebola virus. Results are expressed as Spot-forming units (SFU)/10^6^ splenocytes. Data shown represent the means ± SEM (standard error of the mean) of three different animals. Statistical analysis was made using one-way ANOVA, followed by Tukey multiple comparisons test. Asterisks denote statistically significant data (***P < 0.001, ****P < 0.0001). **b** ZGP IgG responses from immunized groups of mice (*n* = 5/group). Antibodies responses were analyzed by ELISA at day 0 and every week post vaccination from serum samples, which were used in a 1/400 dilution. Results are shown as means ± SEM. Statistical analysis was made using one-way ANOVA, followed by Tukey multiple comparisons test. Asterisks denote statistically significant data (*P < 0.05, **P < 0.01).

To evaluate the immune response induced by vaccination with IONAID, we analyzed the cellular and humoral response of mice following a single immunization and compared this to other methods of vaccination. IONAID was set up to actively inject 100 µl of a pIDV-II plasmid coding for Ebola GP (100 µg total) in the animal back skin. Cellular immune responses were evaluated by ELISpot 10 days after vaccination. ZGP-specific IFN-γ responses increased significantly in animals vaccinated by IONAID when compared to IM injection, where IM-vaccinated animals exhibited 226 ± 30 SFU/10^6^ cells, while IONAID-vaccinated animals exhibited 413 ± 25 SFU/10^6^ cells, representing a 2-fold increase (Fig. 3a). Of note, ID injection was not evaluated in this set of experiments because of its poor performance in inducing ZGP-specific immune responses, as shown above. Humoral immune responses were also evaluated with a ZGP-specific ELISA. On day 28, IgG levels against ZGP in vaccinated mice showed an increase in OD of 3.9-fold in the IONAID group and 1.5-fold in IM group when compared to the control group (Fig. 3b). Moreover, ZGP IgG levels started to increase quickly before day 21 and remained high in the IONAID group. In contrast, ZGP IgG levels in IM-vaccinated animals showed an increase only around day 14, reaching a peak by day 21, and then declined by day 28.

### Impact of tattoo time on the induction of ZGP-specific humoral responses in rabbits

We sought to evaluate whether the enhanced immune responses observed in mice using IONAID were observed also in a larger animal species. New Zealand white rabbits were immunized three times at a two-week interval with 500 µg of pcDNA3.1 plasmid (500 µl at 1 mg/ml) with either IONAID, traditional tattoo delivery (passive tattoo injection), or IM injection. The use of this animal model facilitated the administration of vaccine preparations in a larger area of the skin by DNA tattooing, a limitation with the mouse model. The vaccination procedure reflected that described for mice, except the tattoo site was changed to the inner side of the rabbit ear to facilitate the procedure.

**Fig. 4:**
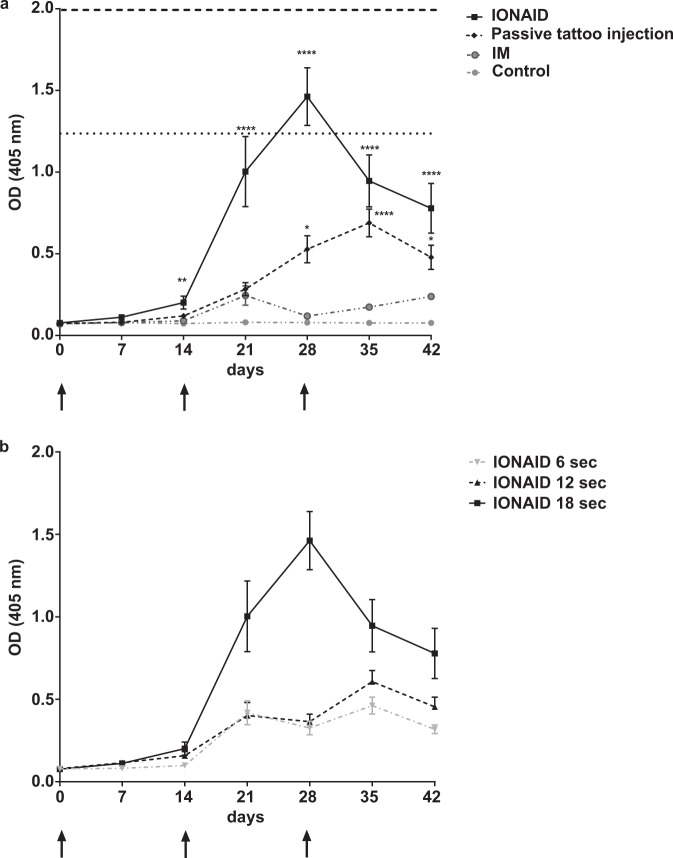
ZGP-enhanced humoral immune responses by IONAID in rabbits. **a** ZGP IgG responses from immunized groups of rabbits (*n* = 3/group) after vaccination with 500 µg of pIDV-II-EboV-GP-M06. Antibodies responses were analyzed by ELISA at day 0 and every week post vaccination from serum samples, which were used in a 1/10,000 dilution. Results are shown as means ± SEM. The arrows indicate the days of the initial vaccination and the two boosters. Horizontal dashed lines show the range of ZGP IgG responses found in Ebola survivors and horizontal dotted lines show the range of ZGP IgG levels found in vaccinated humans. Statistical analysis was made using one-way ANOVA, followed by Tukey multiple comparisons test. Asterisks denote statistically significant data (*P < 0.05, **P < 0.01***, *P* < 0.001, ****P < 0.0001). **b** Influence of the duration of tattooing on ZGP IgG responses from immunized groups of rabbits (*n* = 3/group).

ZGP IgG levels increased quickly after the first vaccination in the IONAID group, peaking at day 28 after the second vaccination (Fig. 4a). At this time point, ZGP IgG levels in the IONAID group were 12-fold higher than IM injection and 2.8-fold higher than passive tattoo injection. Of note, anti-ZGP IgG levels in the IONAID group at this time point were similar to the range of those found in human survivors of Ebola virus disease^19^ (horizontal dashed lines) and higher than those found in ZGP immunized subjects (horizontal dotted lines)^20,21^. Immunization with IONAID substantially increased the humoral responses directed to ZGP, which remained significantly superior to those observed with passive tattoo and IM immunizations. This held true even two weeks after the second boost (day 42), where anti-ZGP IgG levels in the IONAID group were 3.3 higher than in the IM group and 1.6 higher than in the passive tattoo group.

Tattooing produces microlesions on the skin as a result of thousands of needle penetrations, which could have an adjuvant effect to vaccination. The extent of these microlesions depends on the duration of the tattooing procedure, therefore we evaluated the impact of time on the induction of humoral immune responses. Animals were vaccinated as described above. The device was programmed to deliver the same amount of vaccine, 500 µl, but over various lengths of time (6, 12, or 18 s). To achieve this, the valve opening was programed with different injection frequencies; thus, the vaccine injection was every single, second, or third needle oscillation. As shown in Fig. 4b, anti-ZGP IgG levels dropped considerably (4.5-fold lower on day 28 and 2.5-fold lower on day 42, respectively) when the tattoo time was reduced from 18 to 6 s using IONAID.

### The incorporation of a needle guide on IONAID increases consistency in potency

**Fig. 5:**
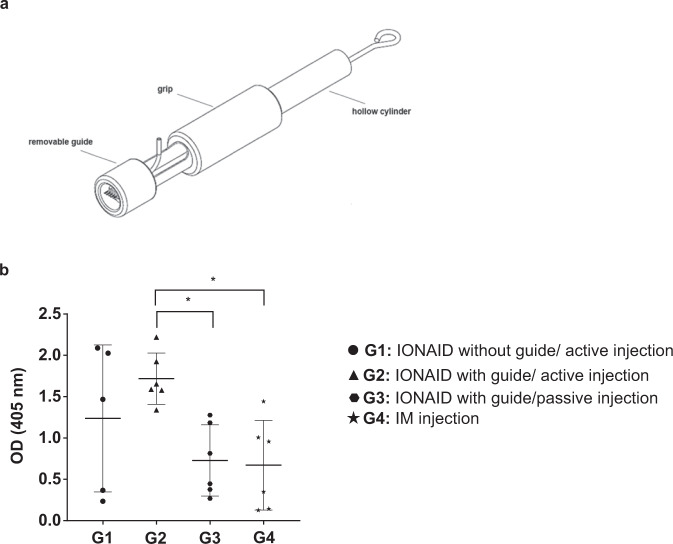
ZGP-enhanced humoral immune responses by IONAID in guinea pigs. **a** Close up of a needle array mounted in the hollow cylinder with the removable plastic guide. **b** ZGP IgG responses from immunized groups of guinea pigs (*n* = 6/group) after vaccination with 300 µg of pIDV-II-EboV-GP-M06. Antibodies responses were analyzed by ELISA at day 35 from serum samples, which were used in a 1/400 dilution. The horizontal line represents the mean of all animals. Vertical lines represent the standard deviation (SD). Statistical analysis was made using one-way ANOVA, followed by Tukey multiple comparisons test. Asterisks denote statistically significant data (*P < 0.05).

Next, we evaluated the humoral immune responses after incorporation of a removable cylindrical plastic guide on the needle array. The purpose of this guide is to regulate the penetration depth of needles in the skin (1–4 mm), keep the vaccine solution close to the needle array, and produce a uniform tattooing (Fig. 5a). Humoral immune responses were compared in vaccinated guinea pigs using IONAID active injection with or without the guide, IONAID without active injection (passive tattoo injection) with guide, or IM injection. IONAID was set up to actively inject 300 µg of a pIDV-II plasmid coding for ZGP (300 µl of 1 mg/ml solution) in the animal back skin. For passive injection with IONAID, the vaccine solution was placed inside the guide before starting the tattooing instead of being actively injected by the needle array. Humoral responses were analyzed at day 35-post vaccination. Anti-ZGP IgG levels were considerably less variable between animals in the IONAID with guide group (G2: active injection, %CV [percent coefficient of variation]: 18.0) compared to IONAID without guide (G1: active injection, %CV: 71.9), IONAID with guide (G3: passive injection, %CV: 59.3) and IM injection (G4: %CV: 80.8) (Fig. 5b and Table 1). As observed in mice and rabbits, anti-ZGP IgG levels in IONAID-vaccinated guinea pigs (active injection) were higher than passive tattoo and IM injections and with less variation using the guide.

**Table 1:** Variability of humoral immune responses after vaccinations.

	Mean O.D.	SD	%CV
G1	1.237	0.889	71.9
G2	1.718	0.310	18.0
G3	0.728	0.431	59.3
G4	0.671	0.542	80.8

Mean, standard deviation (SD), and percent coefficient of variation (%CV) were calculated for each group. G1: IONAID without guide/active injection, G2: IONAID with guide/active injection, G3: IONAID with guide/passive injection, G4: IM injection.

### IONAID immunizations in primates

**Fig. 6:**
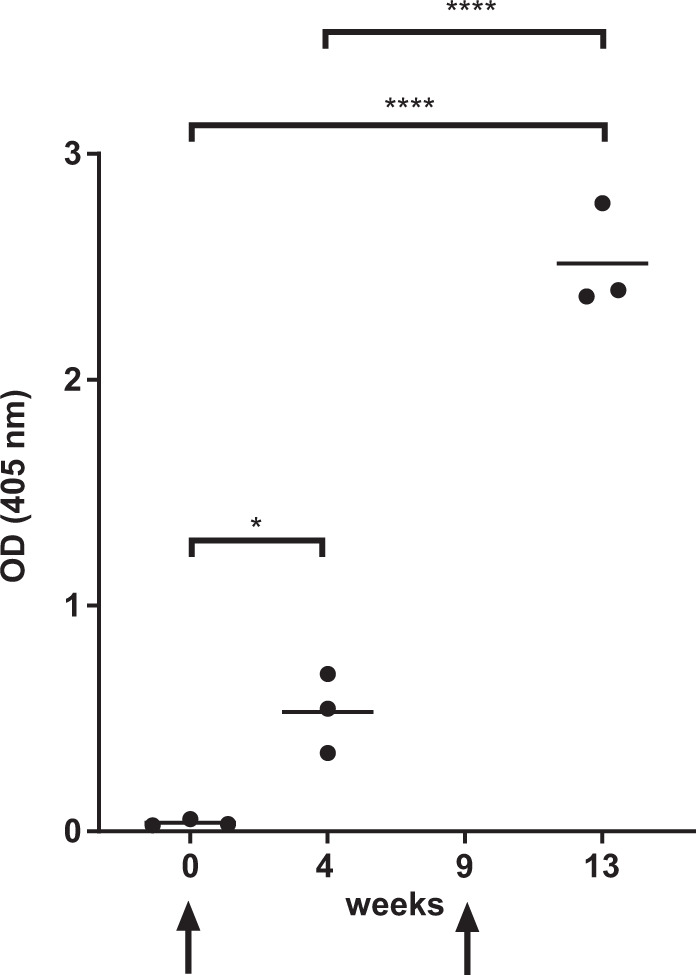
HIV-enhanced humoral immune responses by IONAID boost in non-human primates (NHP). HIV gp140 IgG responses from immunized animals (*n* = 3) after intramuscular vaccination with 1.10^8^ TCID_50_ rVSV-B6-NL4.3Env/Ebotm and boost with 1 mg of pIDV-II-HIVenvNL4.3/Ebotm and 0.3 mg of pIDV-II-revNL4.3 using IONAID. Antibodies responses were analyzed by ELISA at day 0, 4 weeks after vaccination and 4 weeks after boost from serum samples, which were used in a 1/200 dilution. The horizontal line represents the mean of all animals. Statistical analysis was made using one-way ANOVA, followed by Tukey multiple comparisons test. Asterisks denote statistically significant data (*P < 0.05).

Next, we evaluated the safety, tolerability, and the induction of humoral immune responses to another vaccine antigen after boost with IONAID in NHPs. Animals were first vaccinated IM with a recombinant Vesicular Stomatitis-based HIV vaccine (rVSV-B6-NL4.3Env/Ebotm), and 9 weeks later were boosted on the hind limbs using IONAID with a DNA-based HIV vaccine expressing the same HIV antigen (pIDV-II-HIVenvNL4.3/Ebotm/pIDV-II-revNL4.3). The vaccinations were well tolerated, and no adverse events were detected (e.g., no fever, loss of appetite, reduced activity) other than the skin lesion associated with an expected healing process (to a variable extent as small abrasions and inflammation appeared directly after intradermal DNA delivery with IONAID and disappeared within 1 week as commonly seen after regular tattooing). Humoral immune responses were evaluated using an HIV gp140-specific ELISA. A robust increase in anti-gp140 IgG levels was observed after boost with IONAID (4.7-fold higher at week 13 compared to week 4) (Fig. 6). Of note, humoral immune responses remained consistent and reproducible between animals as observed in guinea pigs, presumably from the use of the needle guide.

### Vaccine delivery with IONAID induces neutralizing antibody responses

**Fig. 7:**
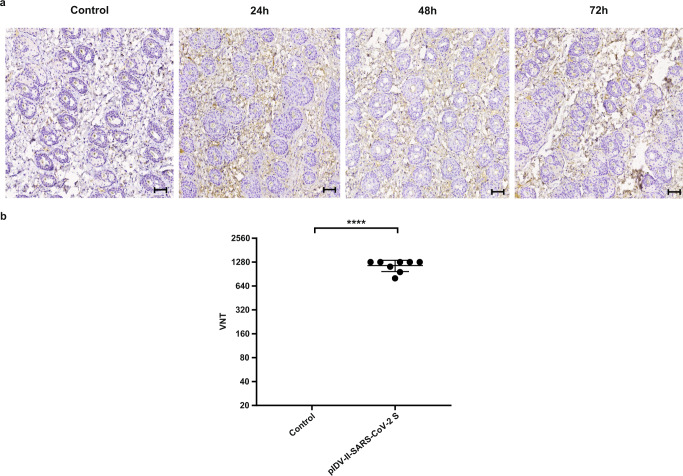
SARS-CoV-2 DNA vaccine delivery by IONAID induces high expression of spike protein and neutralizing antibody responses. **a** Representative immunostaining of the SARS-CoV-2 spike protein in mouse dermis tissues (×10 magnification, scale bar = 100 µm). Skin specimens from mice vaccinated with TE buffer (Control group) or 100 µg of pIDV-II-SARS-CoV-2 Spike (*n* = 3) were analyzed at 24, 48, and 72 h after vaccination. Positive SARS-CoV-2 Spike protein staining is indicated by brown areas. **b** Virus-neutralizing titers (VNT) from hamsters vaccinated with TE buffer (control) or pIDV-II-SARS-CoV-2 Spike were analyzed against SB3 (B1) isolate 4 weeks after booster vaccination. Statistical analysis was made using one-way ANOVA, followed by Tukey multiple comparisons test. Asterisks denote statistically significant data (****P < 0.0001).

Finally, we analyzed protein expression and neutralizing antibody responses after immunization with a different vaccine antigen using IONAID. A pIDV-II plasmid coding for SARS-CoV-2 spike protein was used to monitor protein expression after DNA tattooing with IONAID. First, needle depth was adjusted to 1 mm, and mice were immunized with 100 µg of the SARS-CoV-2 vaccine. Skin specimens of the tattoo sites were dissected at 24 h, 48 h, and 72 h post vaccination. All skin specimens from DNA-vaccinated animals showed positive immunostaining for SARS-CoV-2 protein at all timepoints (Fig. 7a). Next, needle depth was adjusted to 2 mm and the plasmid solution (200 µl at 1 mg/ml) or TE buffer (control group) was applied on the back skin of Syrian hamsters. Then, neutralizing antibody responses against SARS-CoV-2 SB3 (B1) isolate were evaluated 4 weeks after booster vaccination. A robust induction of neutralizing antibodies was observed after boost with IONAID when compared to the control group (Fig. 7b).

## Discussion

Vaccines play an important role in reducing mortality, morbidity, and transmission of infectious diseases. The latest decades have been characterized by a remarkable success of vaccine programs and coverage to curb infectious diseases. However, there is still a need to improve the efficacy of current vaccines and develop new delivery technologies to increase vaccine-mediated protection with the smallest and fewest doses necessary. In this regard, the route of immunization has an important impact in the vaccine efficacy^22^. For several vaccines, various forms of ID delivery have shown to induce superior immune responses when compared to traditional routes of immunization^23–28^. The advantages of ID immunization include the possibility of delivering antigens to a tissue rich in APCs, enhancing the immunogenicity of vaccines, reducing the need for adjuvants, and allowing vaccine dose-sparing. Among the new technologies for ID delivery, DNA tattooing has shown promising results in inducing enhanced immune responses when compared to traditional routes of immunization^12–14^. In line with these studies, we show here that passive DNA tattooing enhances the cellular and humoral immune responses directed to a plasmid expressing ZGP when compared to an IM injection in mice. In contrast, a previous study showed that tattoo immunization provided no immunological advantages in comparison to traditional ID injection of adenoviral vector vaccines^29^. Possibly, the efficacy of tattoo-induced immune responses is specific to different vaccine types, and subsequent delivery protocols should reflect the system with the optimal efficacy.

An explanation for the higher immune responses observed after DNA tattooing could be that the skin, the first barrier against pathogens, is richer in APCs than the muscular tissue, which could provide a significant enhancement to DNA vaccines that are overall less immunogenic than adenovirus-based vaccines^30^. In addition, the thousands of skin perforations produced during tattooing could induce the release of chemokines and cytokines that may serve as potent nonspecific immuno-stimulators (adjuvants)^31^. Skin scarification experiments in rabbits demonstrate the induction of different host transcription profiles compared to ID injection and was shown to confer nonspecific immune response and protection against pox viruses^32^.

The IONAID device was designed to actively inject a defined amount of a pressurized vaccine solution into the skin in a controlled manner. The needle array oscillation is coordinated with the valve opening, thus, during tattooing, the vaccine is injected only when the needles are inside the skin. The antigen expression kinetics induced by IONAID showed peak values of GFP RNA at 24 h and decayed over the next 4 days. Moreover, protein expression was observed 24 h post vaccination with a DNA-based vaccine encoding SARS-CoV-2 spike protein. These results are in line with data obtained in previous studies showing that antigen expression after DNA tattooing occurs over a limited timespan^13,33^. Even so, it has been shown that antigen presentation is significantly better upon tattoo vaccination when compared to IM injection, which provides a likely mechanism for augmented immune responses and dose-sparing^13^. This may impact positively on the development of long-term protective immunity, an important aspect that should be studied in future experiments.

This study shows that IONAID was safe and well tolerated for the administration of a DNA-based HIV vaccine in NHPs. Moreover, the robust humoral immune responses observed in mice, rabbits, and guinea pigs after ZGP vaccine delivery were observed also against HIV GP in NHPs after boosting with the HIV DNA vaccine administered with IONAID. The anticipated added immune stimulation of additional boosts with IONAID in NHPs, as well as a side-by-side comparative analysis to IM injections with or without electroporation, to name a few, requires additional studies. Importantly, the data presented here show a functional antibody response with the generation of high-titers neutralizing antibodies against SARS-CoV-2 in Syrian hamster after IONAID-mediated DNA vaccine delivery. While the experiments above support the enhancement of immune responses when using IONAID over traditional immunization methods, as well as highlighting technical aspects of the vaccination process (duration of vaccination and use of a guide), our group has previously shown IONAID-mediated protection of hamsters against SARS-CoV-2^34^. It would be interesting to compare the performance of the IONAID device to other devices or technologies used for intradermal vaccine delivery, such as EP devices, gene gun, jet injectors, or microneedle patches. Alone, EP has shown promising results in increasing antigen expression and DNA vaccine immunogenicity in animal models and clinical trials^25,35–38^. Furthermore, a combination of tattooing and EP would be feasible with IONAID. This versatile IONAID device has the critical advantages of generating consistent and reproducible immune responses between animals, thus greatly mitigating user variability (Fig. 5b). The proven and recognized dose-sparing effect achieved with ID-vaccine delivery is an untapped strength to reduce the cost of large vaccination campaigns, reach faster global coverage during new epidemics, particularly when vaccine manufacturing cannot meet demand. Currently, this could substantially increase monkeypox vaccine coverage globally through dose-sparing. These advantages, together with the potential to reduce the number of injections and adjuvants needed to develop protective immune responses, are applicable to all vaccines in aqueous solution, whether for human or veterinary use.

The simplicity of IONAID technology allows for quick training, on par with basic tattooing, toward facilitating the rapid global deployment of a vaccine within a population. The device can be operated using single-use disposable needle arrays or, alternatively, the needle arrays can be easily sterilized in a portable steam sterilizer in the field and reused (e.g., for veterinary use). IONAID is relatively simple and inexpensive to produce and could be adapted to be used as a battery-powered transportable device. Still, further engineering and design development should be done on the IONAID prototype to easily scale up the manufacturing process and produce single-use cheap disposable needle arrays or even single-use devices. Thus far, potential disadvantages of IONAID vaccination are the remaining lack of human data and the questions of acceptability in the context of vaccine hesitancy. Three human volunteers who were administered 0.5 or 1 ml of sterile saline reported that IONAID was essentially painless and more like the feeling of scratching. This evaluation, albeit preliminary, bodes well for human vaccination. Additionally favorable for animal vaccination is the commonality of tattooing to identify individuals in a group or herd. This industry ubiquity suggests not only an ease of training, but a potential dual functionality. Finally, it would be informative to evaluate the performance of IONAID in delivering other types of vaccines, such as protein-based, virus like particles (VLP), or viral vector vaccines.

In summary, the data detailed here demonstrate that IONAID outperforms traditional tattooing and IM DNA vaccination in various animal models. This prototype device induced stronger and faster immune responses, improving the delivery, efficacy, and immunogenicity of intradermal-delivered DNA vaccines.

## Methods

### Animals

Female, 6–8-week-old C57BL/6 mice and Syrian hamsters, Hartley guinea pigs weighing ~250–300 g, and 7–9-week-old New Zealand white rabbits were purchased from Charles River. Chinese-origin female rhesus macaques *(Macaca mulatta*) under 10 kg were housed at the Université Laval animal facility. All animals were used and housed in accordance with the Canadian Council on Animal Care guidelines, and all protocols were approved by the Animal Care Ethics Committee of the Université Laval under protocol numbers 2016096-3, 2018042-2, and 2017098-2. Regarding euthanasia, mice and hamsters were anesthetized with 1.5–4% isoflurane before being euthanized by cardiac bleed followed by cervical dislocation; guinea pigs were anesthetized using 1.5–4% isoflurane before being euthanized by cardiac bleed followed by injection of sodium pentobarbital (120 mg/kg); rabbits were sedated with an IM injection of ketamine/xylazine (0.4 mL/kg) + buprenorphine (0.025 mg/kg) before being euthanized by cardiac bleed followed by injection of sodium pentobarbital (50 mg/kg); macaques were sedated by an intramuscular injection of ketamine (10 mg/kg) + buprenorphine (0.01 mg/kg), anesthetized using 3% isoflurane, and euthanized following an IV injection of sodium pentobarbital (120 mg/kg).

### Plasmids and vectors

Plasmids pcDNA3.1-EbovZGP, pIDV-II-EboV-GP-M06, pIDV-II-HIVenvNL4.3/Ebotm, and pIDV-II-eGFP were used in this study. The construction of the pcDNA3.1-EbovZGP was performed using standard cloning techniques, where full-length cDNA encoding the Ebola virus glycoprotein protein (ZGP) was cloned into a CMV promoter-driven mammalian expression vector, pcDNA3.1 (Invitrogen)^39^. pIDV-II-EboV-GP-M06 is a new expression plasmid constructed by our group^40^. To generate this plasmid, ZGP cDNA was amplified from pcDNA3.1-EbovZGP and cloned in pIDV-II plasmid, which is a modified pVAX plasmid containing a Neo/Kanamycin resistance gene for selection in *E. coli*, a CMV promoter fused with chicken β-actin promoter (CAG) for high-level expression in a wide range of mammalian cells, followed with Woodchuck Hepatitis Virus post-transcriptional regulatory element (WPRE) for better regulation of gene expression at the post-transcriptional level. Full cDNA encoding the eGFP protein was also cloned into pIDV-II containing the same transcriptional regulatory elements than pIDV-II-EboV-GP-M06. To generate the rVSV-B6-NL4.3Env/Ebotm vector, the ecto-domain of HIV-1 clone NL4.3 (HIV-1 strain NL4-3 clone SPL7013p44 envelope glycoprotein [env] gene, complete cds: GenBank: JQ975395.1) was fused to Ebola GP transmembrane domain (Ebotm) and cloned into a modified VSVΔG along with an Ebola Zaire GP variant displaying increased tropism for human cells (B6)^41^. The chimeric NL4.3env/Ebotm and NL4.3rev sequences were cloned also into pIDV-II to generate pIDV-II-HIVenvNL4.3/Ebotm and pIDV-II-revNL4.3, respectively. Finally, pIDV-II-SARS-CoV-2 spike-V1 plasmid was generated by performing a sequence alignment of 92 Spike sequences downloaded from NCBI GenBank (Accessed February 24, 2020) and codon-optimized for human use. The gene fragment was synthesized and cloned into pIDV-II^34^.

### Device

The intradermal oscillating needles array injection device (IONAID) comprises a dual-coiled tattoo machine modified with a support to hold an internal housing for receiving a vial containing the vaccine solution (Fig. 1a). A source of compressed and filtered air (200 kPa) flows through the vaccine vial inducing the circulation of the pressurized vaccine through a silicone tube towards a needle array comprised of 11 hollow hypodermic needles (28 gauge) distributed in two parallel rows with their proximal ends connected to an injection head (Fig. 1b). A valve, communicating with the pressurized vaccine path, controls the flow of vaccine solution from the vial down to the needle array. The pressurized vaccine enters the injection head and is evenly distributed into each hypodermic needle. The needle array and injection head are mounted on a traditional tattoo needle with a loop at its extremity, which can be placed on a top hat grommet connected to a tattoo armature bar. Two electromagnetic coils in the tattoo machine move the armature bar with the needle array up and down at ~100 Hz. The valve opening is synchronized with the needle array oscillation, thus the pressurized vaccine is delivered only when the needle array is extended, which in practice confines delivery to inside the skin. The tattoo needles are placed in a regular tattoo hollow cylinder with a grip that serves to hold the machine during tattooing. A control box (not shown) containing a microprocessor and a compressed air cylinder is connected to the tattoo machine and provides the power source. The air pressure in the machine can be regulated with toggles, and a knob on the control box along with the microprocessor controls the valve opening and the precise volume of vaccine to be injected.

### Immunizations

C57BL/6 mice, Syrian hamsters, guinea pigs, rabbits, and rhesus macaques were immunized with 100 µg, 200 µg, 300 µg, 500 µg, or 1 mg of DNA plasmids, respectively, in TE buffer (pcDNA3.1-EbovZGP, pIDV-II-SARS-CoV-2 spike-V1, pIDV-II-EboV-GP-M06, pIDV-II-eGFP or pIDV-II-HIVenvNL4.3/Ebotm: pIDV-II-revNL4.3). For IM injections, DNA plasmids or rVSV-B6-NL4.3Env/Ebotm were administrated into the hind limbs of the animals. For ID injections, the back of the animals were shaved and vaccine preparations were administrated using the Mantoux technique^42^. For traditional tattooing (passive injection) and ID injections using IONAID (active injection), the back of animals (mice and guinea pigs), the inner side of the ear (rabbits), or the hind limbs (rhesus macaques) were shaved and then depilated (Nair^TM^ depilatory cream, Church & Dwight Canada, Inc.). For traditional tattooing, plasmid preparations were applied by pipetting onto the tattooing skin area and the tattooing was performed using an 11-needle array mounted in a two-coil tattoo device (AIMS, Animal Identification and Marking Systems, Inc.). For ID injections using IONAID, the vaccine vial was placed in its support and the pressurized vaccine solution was actively injected inside the skin by the 11-needle array.

Needle depth was adjusted to 1 mm (mouse skin) or 2 mm (Syrian hamster, guinea pig, rabbit, and rhesus macaque skin) for immunizations. After the prime immunization, boosts were conducted at day 28 and 56 in mice experiments, as specified. Boosts were performed at day 14 and 28 in rabbits, at day 28 in Syrian hamsters, and at week 9 in rhesus macaques.

### Analysis of GFP mRNA expression

Mice were immunized with pIDV-II-eGFP using IONAID and then euthanized at 24, 48, 72, or 96 h post-immunization. Mouse-skin specimens were dissected at the tattooing site, treated overnight at 4 °C with RNAlater stabilization solution (ThermoFisher Scientific), and then frozen at −80 °C. Skin samples were placed in a Falcon tube with 300 µl of buffer RLT (Qiagen) and homogenized using Omni Tip Plastic Homogenizer Probes (VWR). The total RNA was isolated from mouse skin using the RNeasy fibrous tissue mini kit according to the manufacturer’s instructions (Qiagen) and was reverse transcribed using SuperScript III Reverse Transcriptase (ThermoFisher Scientific). Transcripts were quantified by real-time PCR using LightCycler 480 SYBR Green I Master Mix (Roche) on a LightCycler 480 Sequence Detector (Roche). GFP and *18* *S* cDNAs were both amplified using the following gene-specific primers: GFP forward (5′-AAGGGCATCGACTTCAAGGA-3′), GFP reverse (5′-GGTGTTCTGCTGGTAGTGGT-3′), 18 S forward (5′-TAGAGGGACAAGTGGCGTTC-3′), and 18 S reverse (5′-CGCTGAGCCAGTCAGTGT-3′). The relative changes in gene expression were calculated using the 2^−ΔΔCt^ method^43^. This method was used after validation experiments demonstrated that the efficiencies of the target and endogenous reference (i.e., *18* *S*) were approximately equal. DNA levels were normalized by quantification of the *18* *S* gene. Results were expressed as fold increase in normalized values over that observed with non-treated skin sections.

### Immunostaining

Mice were immunized with 100 µg of pIDV-II-SARS-CoV-2 spike or TE buffer (control group) using IONAID and then euthanized at 24 h, 48 h, or 72 h post-immunization. Mouse-skin specimens were dissected at the tattooing site and were fixed in 10% neutral phosphate-buffered formalin, routinely processed, and sectioned at 5 μm. Samples were subjected to heat-mediated antigen retrieval with citrate buffer and stained in a 1:1:1:1 ratio for SARS-CoV-2 S protein using the following antibodies, each diluted 1:1000: mouse anti-SARS-CoV S Protein 154C IgM (BEI Resources, Cat. NR-620), 240C IgG2a (BEI Resources, Cat. NR-616), 540C IgG2a (BEI Resources, Cat.NR-618), and 341C IgG2 (BEI Resources, Cat. NR-617) followed by incubation with Mach3 mouse probe (BioCare Medical) and DAB Substrate (BioCare Medical), respectively. Then, the slides were counter-stained with hematoxylin prior to dehydration with 95% and 100% ethanol. Images were acquired on a Huron Digital Pathology Tissue Scope LE slide scanner under the ×10 objective.

### Cellular immune responses

Mice were euthanized at day 10 after the last immunization. Splenocytes from each group of mice were isolated and tested individually. Single-cell suspensions were cleared of red blood cells using ACK lysis buffer (Gibco). Enzyme-Linked Immunospot (ELISpot) assays were conducted according to the manufacturer’s instructions (BD Bioscience, San Jose, California, Cat. 551881). Briefly, 96-well ELISpot plates (Millipore, Billerica, Massachusetts) were coated overnight with anti-mouse interferon γ (IFN-γ) antibody (Ab) (BD Bioscience, San Jose, California, Cat. 51-2525KZ; Diluted according to the manufacturer’s lot-specific recommendation), washed with phosphate-buffered saline (PBS), and blocked with 10% fetal bovine serum (FBS) (Wisent) in Roswell Park Memorial Institute medium (RPMI 1640 [Corning]). A total of 5 × 10^5^ splenocytes in RPMI 10% FBS, 1% Pen/Strep (Wisent), and 2 mM l-glutamine (Life Technologies) were plated per well and stimulated for 18–24 h with 1 µg/mL of a pool of 176 peptides derived from a peptide scan through Envelope glycoprotein (GP/Mayinga-76) of Zaire Ebola virus (JPT, Innovative Peptide Solutions, Berlin, Germany). Cells treated with 1% DMSO in RPMI were used as a negative control, PMA- (10 ng/ml Sigma-Aldrich) and Ionomycin- (500 ng/ml Sigma-Aldrich) treated cells were used as positive controls. The following day, samples were extensively washed before incubation with biotinylated anti-mouse IFN-γ Ab (BD Bioscience, San Jose, California, Cat. 51-1818KA; Diluted according to the manufacturer’s lot-specific recommendation). After incubation with streptavidin–horseradish peroxidase (HRP), IFN-γ–secreting cells were detected using AEC Chromogen (BD biosciences). Finally, spots were counted with an automated AID ELISpot Reader and results were expressed as SFU (spot-forming units)/10^6^ splenocytes.

### Humoral immune responses

Animals were bled 1 day prior to immunization and several weeks after vaccinations. Sera was kept frozen until analyzed. Corning Costar half area 96-well flat-bottom high-binding polystyrene microtiter plates were coated overnight at 4 °C with 30 μl/well of 1 μg/ml recombinant Zaire Ebola virus GP protein (Sino Biological, Inc.) or 2 µg/mL of HIV-1 gp140 recombinant protein (B.6240 gp140C) (NIH HIV reagent program). Plates were blocked for 1 h with blocking buffer (KPL milk diluent/blocking, Sera care [150 μl/well] at 37 °C). Serum was serially diluted in KPL diluent buffer, and 50 μl of the dilution was added to each well and incubated for 1 h at room temperature. The plates were washed six times with PBS–0.1%–Tween 20 (150 μl/well). In all, 50 µl of a secondary HRP-conjugated antibody diluted 1:2000 (goat anti-mouse IgG-HRP [Tonbo; Cat. 72-8093-M001], goat anti-guinea pig IgG-HRP [Tonbo; Cat. 72-8098-M001], goat anti-rabbit IgG-HRP [Tonbo; Cat. 72-8068-M001], or goat anti-human IgG-HRP [Tonbo; Cat. 72-8083-M001) was added to the wells and then incubated for 1 h at 37 °C. The plates were washed six times with PBS–0.1%–Tween 20 (150 μl/well). Horseradish peroxidase substrate (KPL ABTS, Sera care) was then added (50 µl/well) and incubated at 37 °C for 30 min. Reaction was stopped with 50 µl/well of 1% SDS. The plates were read using a Biotek Synergy HTX microplate reader. The data are reported as the optical density at 405 nm (OD405).

### Virus neutralization assay

The virus used for neutralization assays was passage 3 of SARS-CoV-2/SB3-TYAGNC (strain Wuhan), which was isolated from a patient who returned to Canada in March 2020^44^. To evaluate the titers of neutralizing antibodies from vaccinated hamsters, serum specimens were diluted twofold from 1:20 to 1:2560 in DMEM supplemented with 1% penicillin and incubated with 400 TCID50 of the stock virus at 37 °C and 5% CO_2_ for 1 h. After incubation, this mixture was added to 96-well plates containing confluent Vero E6 cells and was incubated at 37 °C and 5% CO_2_ for 1 h. Following this, the liquid overlay was removed and replaced with DMEM containing 2% FBS and 1% P/S. Plates were examined for CPE after 3 and 5 days, and virus neutralization titers (VNT) were recorded as the reciprocal of the highest dilution of serum where the cytopathic effect (CPE) was not observed^45^.

### Statistical analysis

Statistical experimental analyses were performed using the GraphPad Prism software (La Jolla, USA), version 7.04. Statistical analyses were performed using a one-way analysis of variance with Tukey’s multiple comparison post tests. A *P* value ≤0.05 was considered as statistically significant.

### Reporting summary

Further information on research design is available in the Nature Research Reporting Summary linked to this article.

## Supplementary information

Supplementary Information

REPORTING SUMMARY

## Data Availability

The datasets generated and analyzed during the current study are available from the corresponding author upon reasonable request.
