# Supplementary material for: A novel intradermal tattoo-based injection device enhances the immunogenicity of plasmid DNA vaccines
Source: NPJ Vaccines. 2022 Dec 22;7:172. doi: 10.1038/s41541-022-00581-y (PMC9771775; doi:10.1038/s41541-022-00581-y)
Supplement: Supplementary file 1 — Supplementary Information [file 41541_2022_581_MOESM1_ESM.pdf]

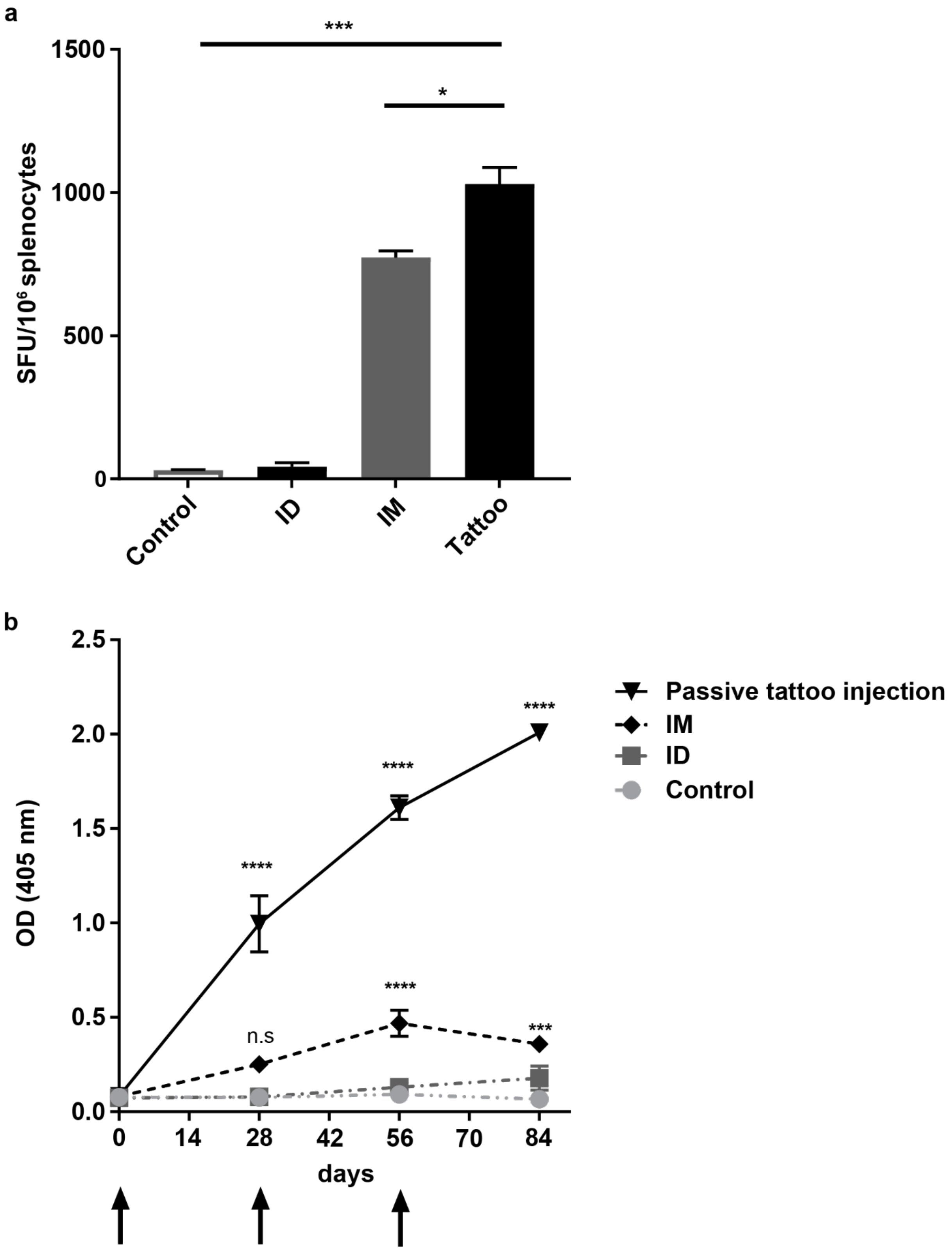

Supplementary Figure 1: ZGP-enhanced immune responses by DNA tattooing in mice. (a) ZGP specific IFN-γ responses in groups of C57BL/6 mice (n=3/group) after

vaccination with 100 µg of pcDNA3.1-GP EBOV Zaire as measured by ELISpot assay. Splenocytes were purified at day 10 after last boost and stimulated with a pool of peptides from GP/Mayinga-76 of Zaire Ebola virus. Results are expressed as Spot forming units (SFU)/106 splenocytes. Data shown represent the means ± SEM of 3 different animals. Statistical analysis was made using one-way ANOVA, followed by Tukey multiple comparisons test. Asterisks denote statistically significant data (*$p < 0.05$, ***$p < 0.001$). **(b)** ZGP IgG responses from immunized groups of mice (n=4/group). Antibodies responses were analyzed by ELISA at day 0 and every 28 days post-vaccination from serum samples, which were used in a 1/1000 dilution. Results are shown as means ± SEM. The arrows indicate the days of the initial vaccination and the two boosters. Statistical analysis was made using one-way ANOVA, followed by Tukey multiple comparisons test. Asterisks denote statistically significant data (***$p < 0.001$, ****$p < 0.0001$).
