# Supplementary material for: A novel intradermal tattoo-based injection device enhances the immunogenicity of plasmid DNA vaccines
Source: NPJ Vaccines. 2022 Dec 22;7:172. doi: 10.1038/s41541-022-00581-y (PMC9771775; doi:10.1038/s41541-022-00581-y)
Supplement: Supplementary file 2 — REPORTING SUMMARY [file 41541_2022_581_MOESM2_ESM.pdf]

nature portfolio

Corresponding author(s): Kobinger, Gary

Last updated by author(s): Aug 12, 2022

Reporting Summary

Nature Portfolio wishes to improve the reproducibility of the work that we publish. This form provides structure for consistency and transparency in reporting. For further information on Nature Portfolio policies, see our Editorial Policies and the Editorial Policy Checklist.

Statistics

For all statistical analyses, confirm that the following items are present in the figure legend, table legend, main text, or Methods section.

n/a	Confirmed	
☐	☒	The exact sample size (*n*) for each experimental group/condition, given as a discrete number and unit of measurement
☐	☒	A statement on whether measurements were taken from distinct samples or whether the same sample was measured repeatedly
☐	☒	The statistical test(s) used AND whether they are one- or two-sided<br>*Only common tests should be described solely by name; describe more complex techniques in the Methods section.*
☒	☐	A description of all covariates tested
☒	☐	A description of any assumptions or corrections, such as tests of normality and adjustment for multiple comparisons
☐	☒	A full description of the statistical parameters including central tendency (e.g. means) or other basic estimates (e.g. regression coefficient) AND variation (e.g. standard deviation) or associated estimates of uncertainty (e.g. confidence intervals)
☐	☒	For null hypothesis testing, the test statistic (e.g. *F*, *t*, *r*) with confidence intervals, effect sizes, degrees of freedom and *P* value noted<br>*Give P values as exact values whenever suitable.*
☒	☐	For Bayesian analysis, information on the choice of priors and Markov chain Monte Carlo settings
☒	☐	For hierarchical and complex designs, identification of the appropriate level for tests and full reporting of outcomes
☒	☐	Estimates of effect sizes (e.g. Cohen's *d*, Pearson's *r*), indicating how they were calculated

Our web collection on statistics for biologists contains articles on many of the points above.

Software and code

Policy information about availability of computer code

Data collection	N/A
Data analysis	GraphPad Prism Version 7.04

For manuscripts utilizing custom algorithms or software that are central to the research but not yet described in published literature, software must be made available to editors and reviewers. We strongly encourage code deposition in a community repository (e.g. GitHub). See the Nature Portfolio guidelines for submitting code & software for further information.

Data

Policy information about availability of data

All manuscripts must include a data availability statement. This statement should provide the following information, where applicable:

- Accession codes, unique identifiers, or web links for publicly available datasets
- A description of any restrictions on data availability
- For clinical datasets or third party data, please ensure that the statement adheres to our policy

The datasets generated and analysed during the current study are available from the corresponding author on reasonable request.

Human research participants

Policy information about studies involving human research participants and Sex and Gender in Research.

Reporting on sex and gender	N/A
Population characteristics	N/A
Recruitment	N/A
Ethics oversight	N/A

Note that full information on the approval of the study protocol must also be provided in the manuscript.

Field-specific reporting

Please select the one below that is the best fit for your research. If you are not sure, read the appropriate sections before making your selection.

☒ Life sciences ☐ Behavioural & social sciences ☐ Ecological, evolutionary & environmental sciences

For a reference copy of the document with all sections, see nature.com/documents/nr-reporting-summary-flat.pdf

Life sciences study design

All studies must disclose on these points even when the disclosure is negative.

Sample size	For animals studies, sample sizes were selected based on mean and SD observed in our previous vaccine studies with these animals models, and the ability to detect statistical diferences between animal groups of this size.<br>In vitro studies were performed with replicates and repeated several times.
Data exclusions	No data was excluded.
Replication	All attempts at replication in the stated conditions were successful.
Randomization	For animal experiments, animals with the same gender, similar age and weight were randomly assigned to each group.
Blinding	N/A

Reporting for specific materials, systems and methods

We require information from authors about some types of materials, experimental systems and methods used in many studies. Here, indicate whether each material, system or method listed is relevant to your study. If you are not sure if a list item applies to your research, read the appropriate section before selecting a response.

Materials & experimental systems

n/a	Involved in the study	
☐	☒	Antibodies
☐	☒	Eukaryotic cell lines
☒	☐	Palaeontology and archaeology
☐	☒	Animals and other organisms
☒	☐	Clinical data
☒	☐	Dual use research of concern

Methods

n/a	Involved in the study	
☒	☐	ChIP-seq
☒	☐	Flow cytometry
☒	☐	MRI-based neuroimaging

Antibodies

Antibodies used	- mouse anti-SARS-CoV S Protein antibodies: 154C IgM, 240C IgG2a, 540C IgG2a, 341C IgG2 (BEI Resources).<br>- goat anti-mouse IgG-HRP, goat anti-guinea pig IgG-HRP, goat anti-rabbit IgG-HRP or goat anti-human IgG-HRP (Tonbo, Bioscience)
Validation	See manufacturer's website for validation of commercial antibodies.

Eukaryotic cell lines

Policy information about cell lines and Sex and Gender in Research

Cell line source(s)	Vero E6 cells: ATCC, CRL-1586.
Authentication	The cell lines were not authenticated since they were purchased commercially and are not commonly misidentified.
Mycoplasma contamination	Cells tested negative for mycoplasma contamination.
Commonly misidentified lines (See ICLAC register)	N/A

Animals and other research organisms

Policy information about studies involving animals; ARRIVE guidelines recommended for reporting animal research, and Sex and Gender in Research

Laboratory animals	Female, 6- to 8-week-old C57BL/6 mice and Syrian hamsters, Hartley guinea pigs weighing ~ 250-300 g, and 7- to 9-week old New Zealand white rabbits were purchased from Charles River. Chinese-origin female rhesus macaques (Macaca mulatta) under 10 kg were housed at the Université Laval animal facility.
Wild animals	Study did not involve wild animals.
Reporting on sex	N/A
Field-collected samples	Study did not involve samples collected from the field.
Ethics oversight	All animals were used and housed in accordance with the Canadian Council on Animal Care guidelines, and all protocols were approved by the Animal Care Ethics Committee of the Université Laval under protocol numbers 2016096-3, 2018042-2 and 2017098-2.

Note that full information on the approval of the study protocol must also be provided in the manuscript.
